# Long non-coding RNA cancer susceptibility candidate 2 (CASC2) alleviates the high glucose-induced injury of CIHP-1 cells via regulating miR-9-5p/PPARγ axis in diabetes nephropathy

**DOI:** 10.1186/s13098-020-00574-8

**Published:** 2020-08-06

**Authors:** Feng Li, Bo Dai, Xiquan Ni

**Affiliations:** 1 Department of Nephrology, Heze Mudan People’s Hospital, Heze, Shandong China; 2 Department of Nephrology, Liaocheng People’s Hospital, Liaocheng, Shandong China; 3 Department of Nephrology, Tai’an Campus of the 960th Hospital of the Chinese People’s Liberation Army, No.217 Huanshan Road, Taishan District, Tai’an, 271000 Shandong China

**Keywords:** High glucose, Podocyte, CASC2, miR-9-5p, PPARγ

## Abstract

**Background:**

High glucose (HG) induced podocytes injury plays an important role in diabetes nephropathy (DN) development. Long noncoding RNA cancer susceptibility candidate 2 (CASC2) was found to be decreased in serum of DN patients. We aimed to explore the function and possible mechanism of CASC2 in HG induced podocytes injury.

**Methods:**

Under normal glucose (NG), HG and mannitol stimulated podocyte conditions, the levels of CASC2, microRNA-9-5p (miR-9-5p) and peroxisome proliferator-activated receptor gamma (PPARγ) were examined by quantitative real-time polymerase chain reaction (qRT-PCR). Podocyte injury was evaluated by measuring cell viability and apoptosis of CIHP-1 cells were checked by cell counting kit-8 (CCK-8) assay and flow cytometry, respectively. Western blot was used to detect all protein levels. Dual-luciferase reporter, RNA immunoprecipitation (RIP) and RNA pull-down assays were performed to confirm the relationship between CASC2 and miR-9-5p.

**Results:**

HG stimulation inhibited the expression levels of CASC2 and PPARγ, but promoted the expression of miR-9-5p. HG could restrain cell viability, autophagy and facilitate apoptosis in CIHP-1 cells, while CASC2 overexpression could reverse HG-induced podocytes injury. Furthermore, CASC2 could be used as a ceRNA to adsorb miR-9-5p, and miR-9-5p mimic overturned the effects of CASC2 on cell viability, autophagy and apoptosis in HG-stimulated podocytes. Additionally, PPARγ was a target gene of miR-9-5p, and CASC2 could weaken the HG-induced podocytes injury by up-regulating PPARγ.

**Conclusion:**

CASC2 increased cell viability, autophagy and inhibited cell apoptosis by regulating miR-9-5p/PPARγ axis, thus reducing the HG-induced podocytes injury.

## Background

Diabetes is a common endocrine disease, among which the prevalence of diabetes nephropathy (DN) is 20–40% [1]. It is estimated that the number of DN patients is expected to increase to 642 million by 2040 [2]. DN is characterized by the presence of albuminuria and a decreased glomerular filtration rate [3]. Podocyte cells (podocytes) are epithelial cells in the visceral layer of renal follicles, which play a key role in the pathogenesis of DN and are an important component of glomerular filtration barrier [4, 5]. Several studies have revealed the correlation between podocytes injury (death and apoptosis) and albuminuria [6], and reducing podocyte injury can improve DN [7]. However, the mechanism for alleviating podocytes injury remains unclear.

Long non-coding RNAs (lncRNAs), are non-protein-coding RNA molecules longer than 200 nucleotides, which are widely regarded as the important regulators in cellular function and disease processes [8]. Increased evidences suggested that lncRNA could modulate DN progression. For instance, lncRNAs GM5524 and GM15645 could regulate the HG-stimulated podocyte autophagy in DN [9]. LncRNA PVT1 knockdown repressed podocytes injury and apoptosis via increasing FOXA1 [10]. However, there are still many lncRNAs in DN function and molecular mechanisms have not been studied.

LncRNA cancer susceptibility candidate 2 (CASC2), located on chromosome 10q26, plays a regulatory role as an anti-cancer factor in various cancers, such as hepatocellular carcinoma [11] and pancreatic carcinoma [12]. Recently, Wang et al. revealed that CASC2 was specifically reduced in serum and renal tissues of type 2 diabetes patients with chronic renal failure, and follow-up identified that the serum of patients with low CASC2 expression had higher incidence of chronic renal failure [13]. MicroRNA-9-5p (miR-9-5p) is both a tumor depressor and a tumor promoter [14, 15]. A report demonstrated that miR-9-5p was related to complications of nephropathy in Type 1 and Type 2 diabetes patients [16]. The mechanism by which lncRNA can serve as the competing endogenous RNA (ceRNA) for miRNA to modulate the abundance of mRNA has been widely reported [17]. Peroxisome proliferator-activated receptor gamma (PPARγ) is implicated in several metabolic syndromes, including DN. Down-regulated PPARγ could activate β-catenin signaling to destroy podocyte architectural integrity and increase cell apoptosis in DN [18]. Furthermore, lncRNA TUG1 could relieve extracellular matrix accumulation by sponging miR-377 and regulating PPARγ in DN [19]. Based on the above findings, we speculated whether CASC2 can modulate PPARγ expression by serving as a ceRNA of miR-9-5p in DN.

In this work, we aimed to explore the effects of CASC2 on cell viability, apoptosis and autophagy in high glucose (HG) induced podocytes, and probe the relationship among CASC2, miR-9-5p and PPARγ, providing a new perspective on the molecular mechanism of podocytes injury in DN.

## Materials and methods

### Cell culture and high glucose induction

Human podocytes CIHP-1 (Ximbio, London, USA) were cultured in a Dulbecco’s modified Eagle’s medium (DMEM, Invitrogen, Carlsbad, CA, USA) containing 10% fetal bovine serum (FBS, Gibco, Carlsbad, CA, USA) at a temperature of 37 °C with 5% CO_2_. When cells density reached about 70%, CIHP-1 cells were exposed to normal glucose (NG, 5 mM), high glucose (HG, 30 mM) or mannitol (30 mM), and the exposure time was determined by individual experiments required.

### Cell transfection

CASC2 overexpressed plasmid (CASC2) and its control Vector, small interfering RNAs against CASC2 and PPARγ (si-CASC2, si-PPARγ) and matched si-NC were provided by GenePharma (Shanghai, China). miR-9-5p mimic (miR-9-5p), inhibitor (anti-miR-9-5p) and their corresponding references (miR-NC, anti-NC) were synthesized by Beyotime (Beijing, China). Transfection of podocytes was performed by using Lipofectamine 3000 (Invitrogen).

### Quantitative real-time polymerase chain reaction (qRT-PCR)

The RNA in CIHP-1 cells was extracted by TRIzol (Invitrogen), and the complementary DNA (cDNA) was synthesized via reverse transcription using HiScript Q RT Super Mix (Vazyme, Piscataway, NJ, USA). The reverse transcription was performed at 42 °C for 10 min and at 85 °C for 10 s. qRT-PCR analysis was conducted on 7500 Real-Time PCR System (Applied Biosystems, Foster City, CA, USA) using the SYBR premix Ex TaqIIkit (TaKaRa, Wuhan, China). Glyceraldehyde-3-phosphate dehydrogenase (GAPDH) and U6 were used as endogenous controls for CASC2/PPARγ and miR-9-5p, respectively. The primers used in this paper were synthesized by GenePharma and the sequences were used as below: CASC2, forward (F) 5′-GCACATTGGACGGTGTTTCC-3′, reverse (R) 5′-CCCAGTCCTTCACAGGTCAC-3′. miR-9-5p, F 5′-GTGCAGGGTCCGAGGT-3′, R 5′-GCGCTCTTTGGTTATCTAGC-3′. PPARγ, F 5′-AGAGCCTTCCAACTCCCTCA-3′, R 5′-AACAGCTTCTCCTTCTCGGC-3′. U6, F 5′-TTGGTGCTCGCTTCGGCA-3′, R 5′-GTGCAGGGTCCGAGGT-3′. GAPDH, F 5′-GGAGTCCACTGGTGTCTTCA-3′, R 5′-GGGAACTGAGCAATTGGTGG-3′.

### Cell viability and apoptosis detection

CIHP-1 cells were tiled into the 96-well plates and exposed to different treatments (HG, NG, HG + Vector, HG + CASC2 and so on). At given points in time (12 h, 24 h and 48 h), 10 µL cell counting kit-8 (CCK-8, Beyotime) was added to the cells and cultured for another 2 h at 37 °C. Finally, the absorbance at 450 nm was measured by Biotek-Epoch2 (Beijing, China).

The apoptosis of podocytes CIHP-1 was estimated at 48 h after exposure to different treatments by using an Annexin V fluorescein isothiocyanate (FITC) and propidium iodide (PI) apoptosis detection kit (Keygen, Beijing, China). Briefly, podocytes were collected and were then suspended in 5 µL FITC and 5 µL PI in the absence of light for 15 min. The apoptosis of CIHP-1 cells was checked by a flow cytometer (BD Biosciences, Franklin Lake, NJ, USA)

### Western blot assay

Total protein from CIHP-1 cells was extracted by RIPA (Beyotime), and denatured at 98 °C for 7 min before separation, and then transferred to polyvinylidene difluoride (PVDF, Beyotime) membranes. Membranes were sealed with 5% milk for 2 h before incubation with primary antibodies against B-cell lymphoma-2 (BCL-2, 1:1000, Abcam, Cambridge, MA, USA), Cleaved-caspase-3 (1:500, Abcam), Light chain 3-II (LC3-II, 1:3000, Abcam), LC3-I (1:1000, Abcam), Beclin 1 (1:2000, Beyotime), PPARγ (1:500, Abcam) or GAPDH (1:2000, Beyotime) overnight at 4 °C. HRP-conjugated secondary antibody (1:4000, Abcam) was employed to incubate the membranes for another 1 h. And the proteins were visualized by using BeyoECL Moon (Beyotime).

### Dual-luciferase reporter assay

CASC2 wild type (CASC2-wt) with miR-9-5p binding sites and its mutant type (CASC2-mut) without binding sites were co-transfected into CIHP-1 cells with miR-9-5p or miR-NC, respectively. Transfection was continued for 36 h and luciferase activity was evaluated through a Dual-luciferase reporter kit (Promega, Madison, WI, USA). In the same manner, PPARγ 3′untranslated region (3′UTR)-wt with miR-9-5p binding sites and PPARγ 3′UTR-mut were co-transfected into cells with miR-9-5p or miR-NC, respectively, and the luciferase activity was detected.

### RNA immunoprecipitation (RIP) assay and RNA pull-down assay

RIP detection was conducted using a Magna RIP RNA-Binding Protein Immunoprecipitation Kit (Millipore, Billerica, MA, USA). CIHP-1 cells were treated with miR-9-5p or miR-NC, 48 h later, cells were lysed in RIP Lysis Buffer containing protease inhibitors. Then, Argonaute2 (Ago2) or ImmunoglobulinG (IgG) antibody (Abcam) were added to the cell lysates overnight at 4 °C, and the immunoprecipitated RNAs were obtained, CASC2 and miR-9-5p levels were estimated using qRT-PCR analysis.

CIHP-1 cells were transfected with Biotin labeled Bio-miR-9-5p and Bio-miR-NC, respectively. At 48 h post-transfection, cells were collected and the bound RNA was obtained by using a Pierce™ Magnetic RNA Pull-Down Kit (Thermo Fisher Scientific, Waltham, MA, USA) according to the instructions. Finally, CASC2 enrichment was assessed by qRT-PCR.

### Statistical analysis

Data were acquired from at least three independent repetitions and displayed as mean ± standard deviation (SD). Difference analysis was conducted by Student’s *t*-test with two groups and one-way analysis of variance (ANOVA) with multiple groups using GraphPad Prism 8. The *P* value less than 0.05 was regarded as statistically distinct.

## Results

### CASC2 alleviated the HG-induced podocytes injury

**Fig. 1:**
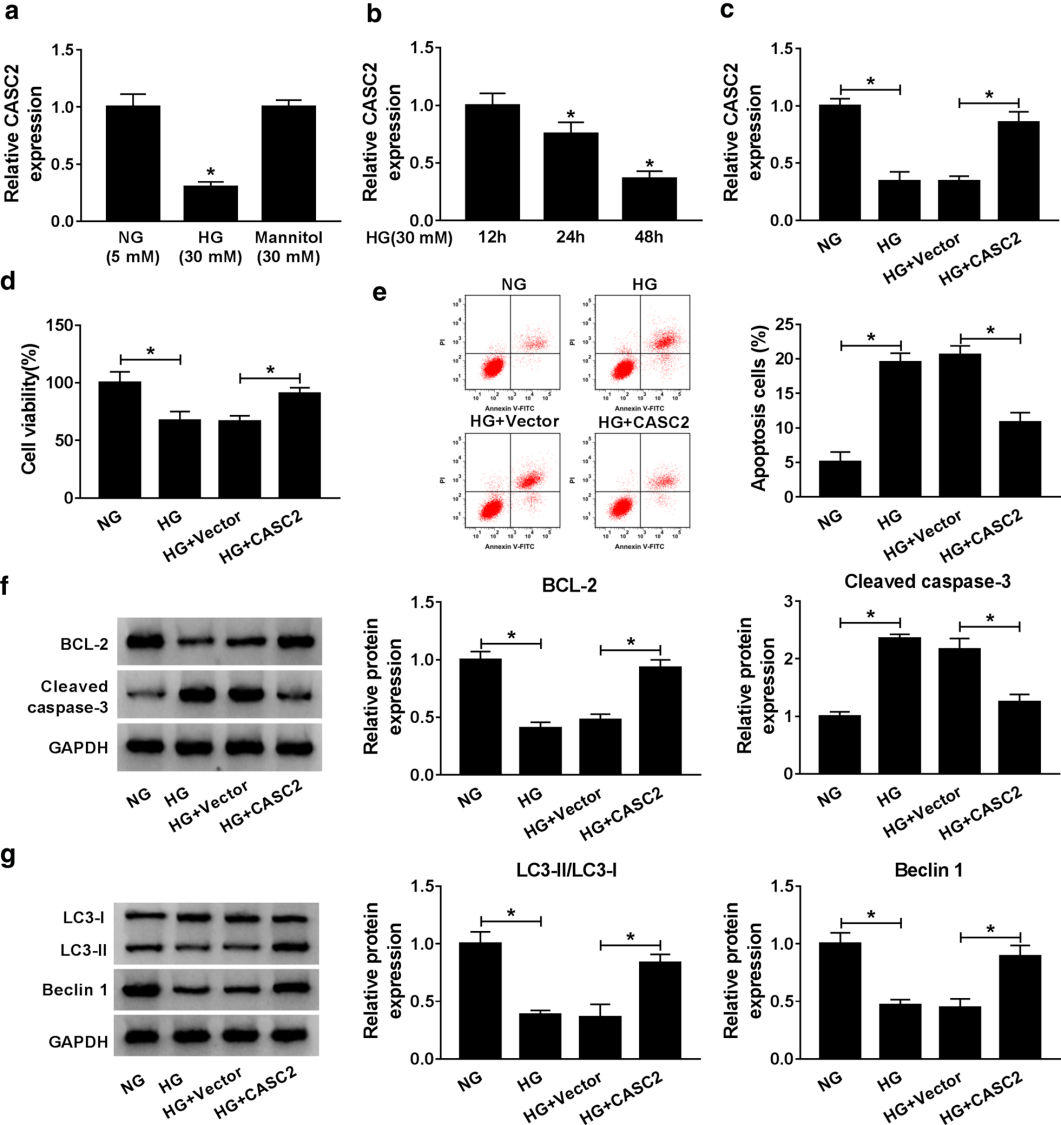
CASC2 alleviated the HG-induced podocytes injury. **a** The expression of CASC2in CIHP-1 cells treated with normal glucose (NG), high glucose (HG) or mannitol was detected by qRT-PCR. **b** After CIHP-1 cells were treated with HG (mM) for 12 h, 24 h and 48 h, respectively, CASC2 expression was measured by qRT-PCR. **c** CIHP-1 cells were divided into four groups, which were control, NG (5 mM),  HG (30 mM), HG  + vector and HG + CASC2, CASC2 expression was detected by qRT-PCR. **d** Cell viability was assessed by CCK-8 assay. **e** Cell apoptosis was examined by flow cytometry. **f**, **g** Western blot assay was used to determine the expression levels of apoptosis-related proteins BCL-2 and Cleaved-caspase-3 and autophagy related proteins LC3-II, LC3-I and Beclin 1. *P < 0.05

Firstly, we examined the expression of CASC2 in human podocytes treated with NG, HG or mannitol by qRT-PCR. The results showed that HG significantly decreased CASC2 expression in CIHP-1 cells compared with NG and mannitol treatment (Fig. 1a). In addition, a time-dependent reduction in CASC2 expression was displayed in HG-treated CIHP-1 cells (12, 24 and 48 h) (Fig. 1b). In view of the expression of CASC2 was substantially reduced at 48 h of HG stimulation, we then overexpressed CASC2 in HG-stimulated CIHP-1 cells for 48 h, and overexpression efficiency was identified by qRT-PCR. As shown in Fig. 1c, CASC2 expression was obviously promoted in HG-stimulated CIHP-1 cells after transfection of CASC2 for 48 h. CCK-8 and flow cytometry results indicated that overexpression of CASC2 induced cell viability (Fig. 1d) and retarded apoptosis (Fig. 1e) in HG-treated CIHP-1 cells. To confirm the results of apoptosis, we detected the expression of apoptosis marker proteins BCL-2 and Cleaved-caspase-3. Western blot assay demonstrated that up-regulation of CASC2 enhanced BCL-2 expression and silenced Cleaved-caspase-3 expression (Fig. 1f), which was in agreement with the results of Annexin V-FITC/PI. Furthermore, HG could reduce the ratio of LC3-II/LC3-I and Beclin 1 expression in CIHP-1 cells, and CASC2 overexpression reversed the effects of HG on the expression of autophagy related proteins (Fig. 1g). The above findings indicated that CASC2 could alleviate the HG-induced podocytes injury by affecting cell viability, apoptosis and autophagy.

### CASC2 directly interacted with miR-9-5p

**Fig. 2:**
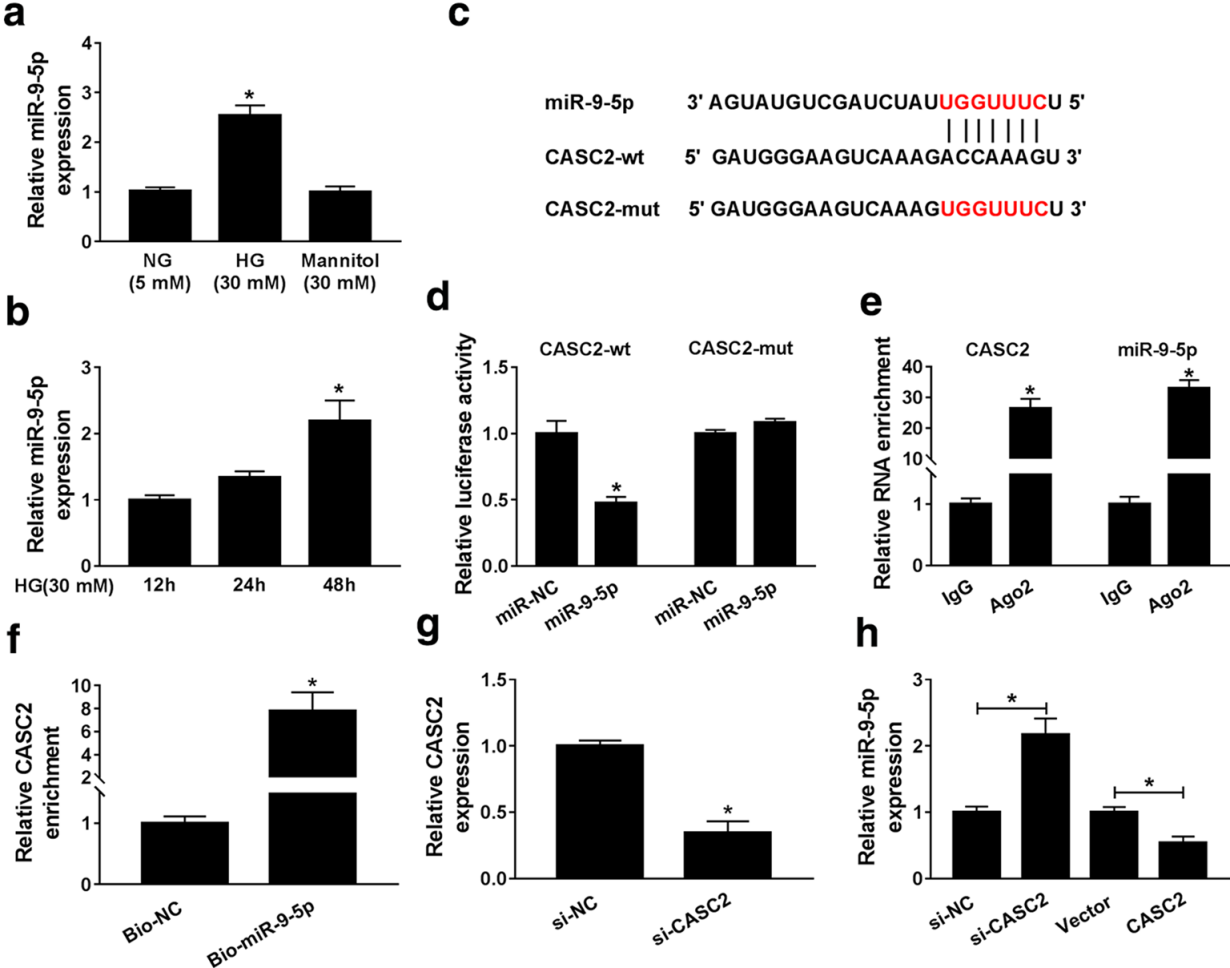
CASC2 directly interacted with miR-9-5p. **a** The expression of miR-9-5pin CIHP-1 cells treated with normal glucose (NG), high glucose (HG) or mannitol was measured by qRT-PCR. **b** After CIHP-1 cells were treated with HG (mM) for 12 h, 24 h and 48 h, respectively, miR-9-5p expression was examined by qRT-PCR. **c** StarBase v2.0 was used to predict the target miRNAs of CASC2. **d**–**f** Dual luciferase reporter, RIP and RNA pull-down assays were utilized to assess the combination of CASC2 and miR-9-5p. **g** CASC2 expression in CIHP-1 cells transfected with si-NC or si-CASC2 was determined by qRT-PCR. **h** The expression of miR-9-5pin CIHP-1 cells transfected with si-NC, si-CASC2, Vector or CASC2 was measured using qRT-PCR analysis. *P < 0.05

LncRNA generally functions as a sponge for miRNA in human diseases [20]. We speculated whether CASC2 could also act as miRNA sponge to regulate HG-induced podocytes injury. As shown in Fig. 2a, we found that miR-9-5p was up-regulated in HG-treated CIHP-1 cells compared to cells treated with NG or mannitol, and miR-9-5p expression was drastically augmented in HG-treated CIHP-1 cells in a time-dependent manner (Fig. 2b). Interestingly, there were complementary sites between miR-9-5p and CASC2 by bioinformatics website starBase v2.0 (Fig. 2c). Dual-luciferase reporter assay showed that the luciferase activity of CASC2-wt was obviously decreased in CIHP-1 cells transfected with miR-9-5p than that cells transfected with miR-NC, whereas, it was no significant difference in luciferase activity of CASC2-mut (Fig. 2d). RIP assay indicated that the enrichments of CASC2 and miR-9-5p were higher in CIHP-1 cells incubated with Ago2 (Fig. 2e). RNA pull-down assay further revealed that the enrichment of CASC2 in Bio-miR-9-5p group was aggrandized relative to that Bio-NC group (Fig. 2f). These results strongly supported that CASC2 could specifically bind to miR-9-5p. Meanwhile, qRT-PCR data showed that CASC2 knockdown in CIHP-1 cells elevated miR-9-5p expression, and CASC2 overexpression degraded miR-9-5p expression (Fig. 2g, h). These results suggested that CASC2 could act as a ceRNA to negatively regulated miR-9-5p expression in podocytes.

### CASC2 regulated the HG-induced podocytes injury via targeting miR-9-5p

**Fig. 3:**
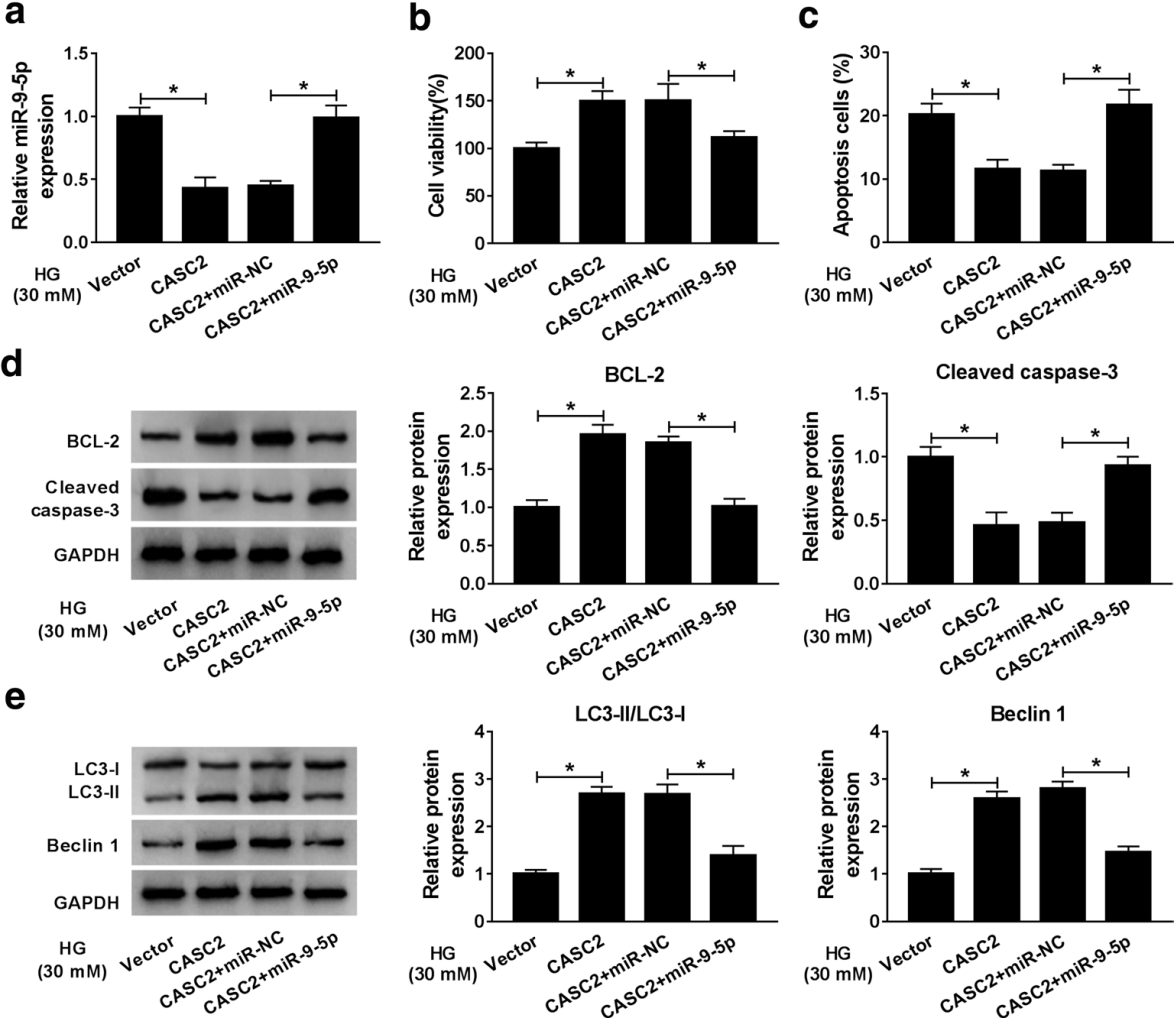
CASC2 regulated the HG-induced podocytes injury via targeting miR-9-5p. The HG-treated CIHP-1 cells were divided into four groups: Vector, CASC2, CASC2 + miR-NC and HG + miR-9-5p. **a** The expression of miR-9-5p was examined by qRT-PCR. **b**, **c** Cell viability and apoptosis were evaluated by CCK-8 assay and flow cytometry, respectively. **d**, **e** The expression levels of apoptosis-related proteins BCL-2 and Cleaved-caspase-3 and autophagy related proteins LC3-II, LC3-I and Beclin 1 were detected by western blot assay. *P < 0.05

As presented in Fig. 3a, miR-9-5p mimic (miR-9-5p) could reverse the inhibitory effect of CASC2 overexpression on miR-9-5p expression in HG-induced CIHP-1 cells. As expected, the impact of CASC2 on promoting cell activity (Fig. 3b) and inhibiting cell apoptosis (Fig. 3c) in HG-stimulated CIHP-1 cells was offset by miR-9-5p. Simultaneously, the inhibition of CASC2 on the protein expression of Cleaved-caspase-3 and the promotion of CASC2 on LC3-II/LC3-I ratio as well as the levels of BCL-2 and Beclin 1 could be weakened by transfection of miR-9-5p in HG-induced CIHP-1 cells (Fig. 3d, e). The obtained data proved that CASC2 attenuated the HG-induced podocytes injury by down-regulating miR-9-5p.

### CASC2 acted as a ceRNA by sponging miR-9-5p to facilitate PPARγ expression

**Fig. 4:**
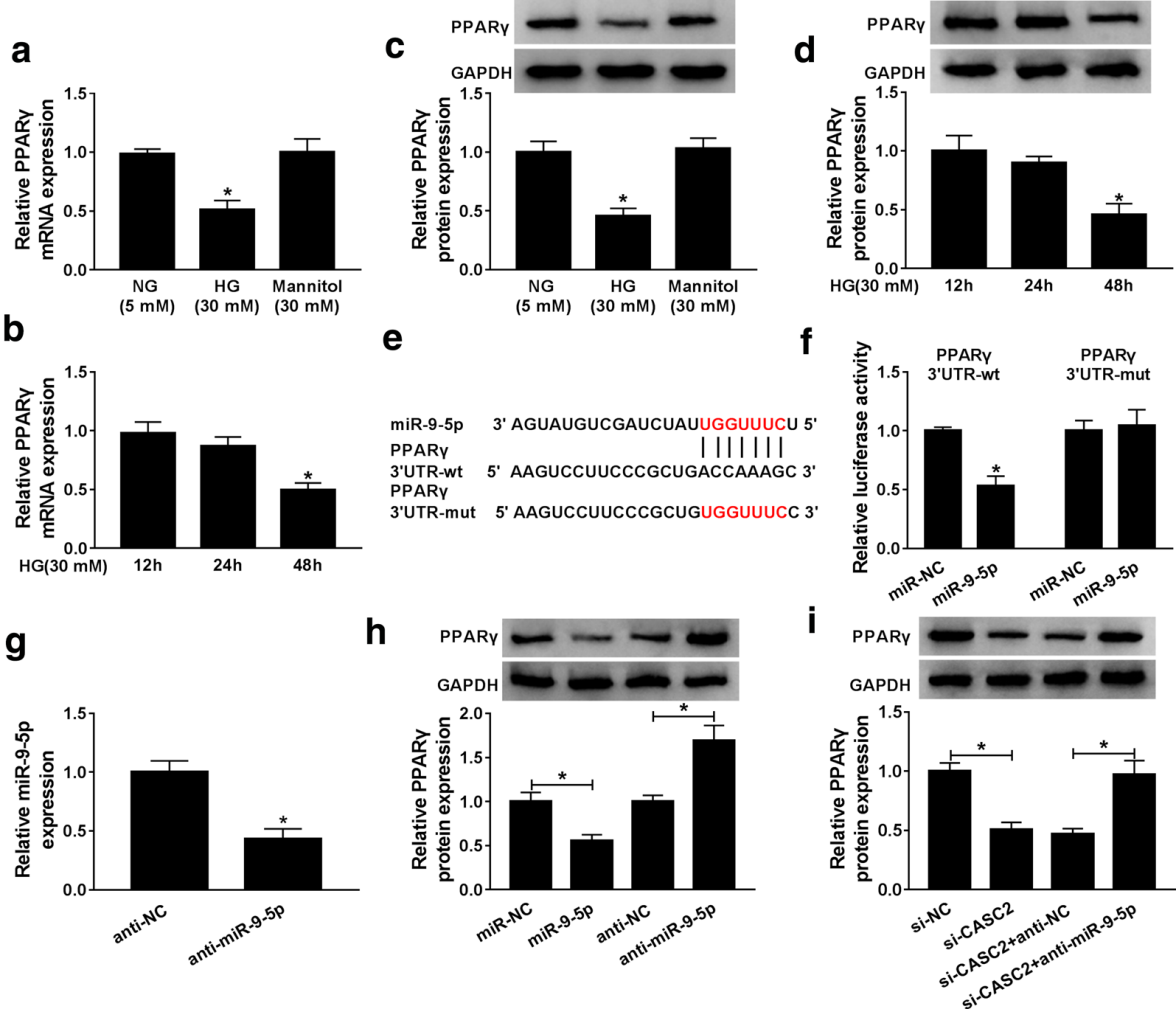
CASC2 acted as a ceRNA by sponging miR-9-5p to facilitate PPARγ expression. **a** The mRNA expression of PPARγ in NG, HG or mannitol-treated CIHP-1 cells was analyzed by qRT-PCR. **b** qRT-PCR assay was used to measure the mRNA expression of PPARγ in CIHP-1 cells treated by HG (30 mM) at different times. **c** The protein expression of PPARγ in NG, HG or mannitol-treated CIHP-1 cells was analyzed by western blot assay. **d** Western blot assay was used to measure the protein expression of PPARγ in CIHP-1 cells treated by HG (30 mM) at different times. **e** StarBase v2.0 predicted that there were binding sites between miR-9-5p and PPARγ. **f** Dual luciferase reporter assay was conducted to detect the interaction between miR-9-5p and PPARγ in CIHP-1 cells. **g** The expression of miR-9-5p in CIHP-1 cells transfected with anti-NC or anti-miR-9-5p was measured by qRT-PCR. **h** The protein expression of PPARγ in CIHP-1 cells transfected with miR-NC, miR-9-5p, anti-NC or anti-miR-9-5p was assessed using western blot assay. **i** PPARγ protein expression in CIHP-1 cells transfected with si-NC, si-CASC2, si-CASC2 + anti-NC, or si-CASC2 + anti-miR-9-5p was estimated by western blot. *P < 0.05

As appeared in Fig. 4a–d, HG inhibited the mRNA and protein levels of PPARγ in CIHP-1 cells compared to NG and mannitol stimulation. At 48 h after the induction of HG, the mRNA and protein levels of PPARγ were dwindled in CIHP-1 cells. The effect of HG treatment on PPARγ expression was the opposite of that of miR-9-5p, thus we speculated whether there was a connection between miR-9-5p and PPARγ. As presented in Fig. 4e, there were binding sites for miR-9-5p in the 3′UTR of PPARγ. Dual-luciferase reporter assay showed that miR-9-5p markedly decreased the luciferase activity of PPARγ 3′UTR-wt in CIHP-1 cells than that PPARγ 3′UTR-mut (Fig. 4f), suggesting PPARγ was the target mRNA of miR-9-5p. Then, we examined the effect of miR-9-5p on PPARγ expression, the interference efficiency of anti-miR-9-5p on miR-9-5p expression was first examined by qRT-PCR (Fig. 4g). Western blot data showed that the overexpressed miR-9-5p could restrain the protein expression of PPARγ, while the decreased miR-9-5p could raise PPARγ protein expression (Fig. 4h). Additionally, we found that CASC2 depletion reduced the protein expression of PPARγ, and co-transfection of anti-miR-9-5p could reverse this effect (Fig. 4i). The above findings revealed that CASC2 positively regulated PPARγ expression by acting as a ceRNA for miR-9-5p in podocytes.

### CASC2 alleviated the HG-induced podocytes injury by increasing PPARγ

**Fig. 5:**
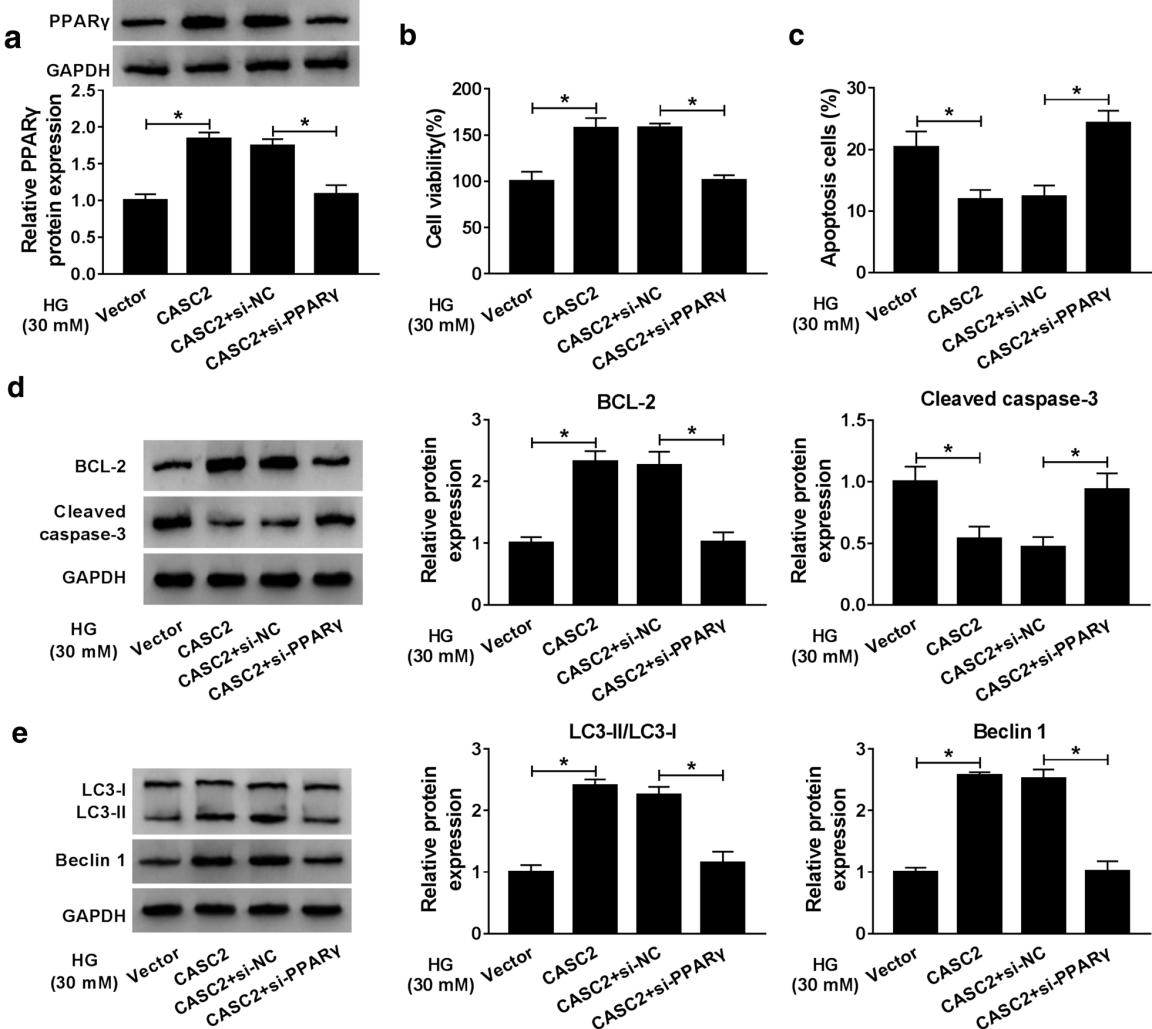
CASC2 alleviated the HG-induced podocytes injury by increasing PPARγ. The HG-treated CIHP-1 cells were transfected with Vector, CASC2, CASC2 + si-NC and si-PPARγ, respectively. **a** PPARγ protein expression was examined by western blot. **b**, **c** Cell viability and apoptosis were determined by CCK-8 assay and Flow cytometry, respectively. **d**, **e** The expression levels of BCL-2, Cleaved-caspase-3, LC3-II, LC3-I and Beclin 1 were checked by western blot assay. *P < 0.05

Considering CASC2 could act as a sponge of miR-9-5p to regulate the expression of PPARγ, we further investigated whether PPARγ was involved in regulation of HG-induced podocytes injury mediated by CASC2. Western blot results indicated that co-transfection of si-PPARγ neutralized the promoting effect of CASC2 on PPARγ protein expression (Fig. 5a). The data of CCK-8 and Annexin V-FITC/PI assays indicated that the effects of CASC2 on cell viability (Fig. 5b) and apoptosis (Fig. 5c) could be abolished by silencing PPARγ in HG-induced CIHP-1 cells. Similarly, the effects of CASC2 on levels of Cleaved-caspase-3, BCL-2, Beclin 1 and LC3-II/LC3-I ratio were rescued by si-PPARγ, implying PPARγ knockdown could increase Cleaved-caspase-3 protein, and decrease the expression levels of BCL-2 and Beclin 1 as well as the ratio of LC3-II/LC3-I (Fig. 5d, e). To sum up, CASC2 alleviated the HG-induced podocytes injury by up-regulating PPARγ.

**Fig. 6:**
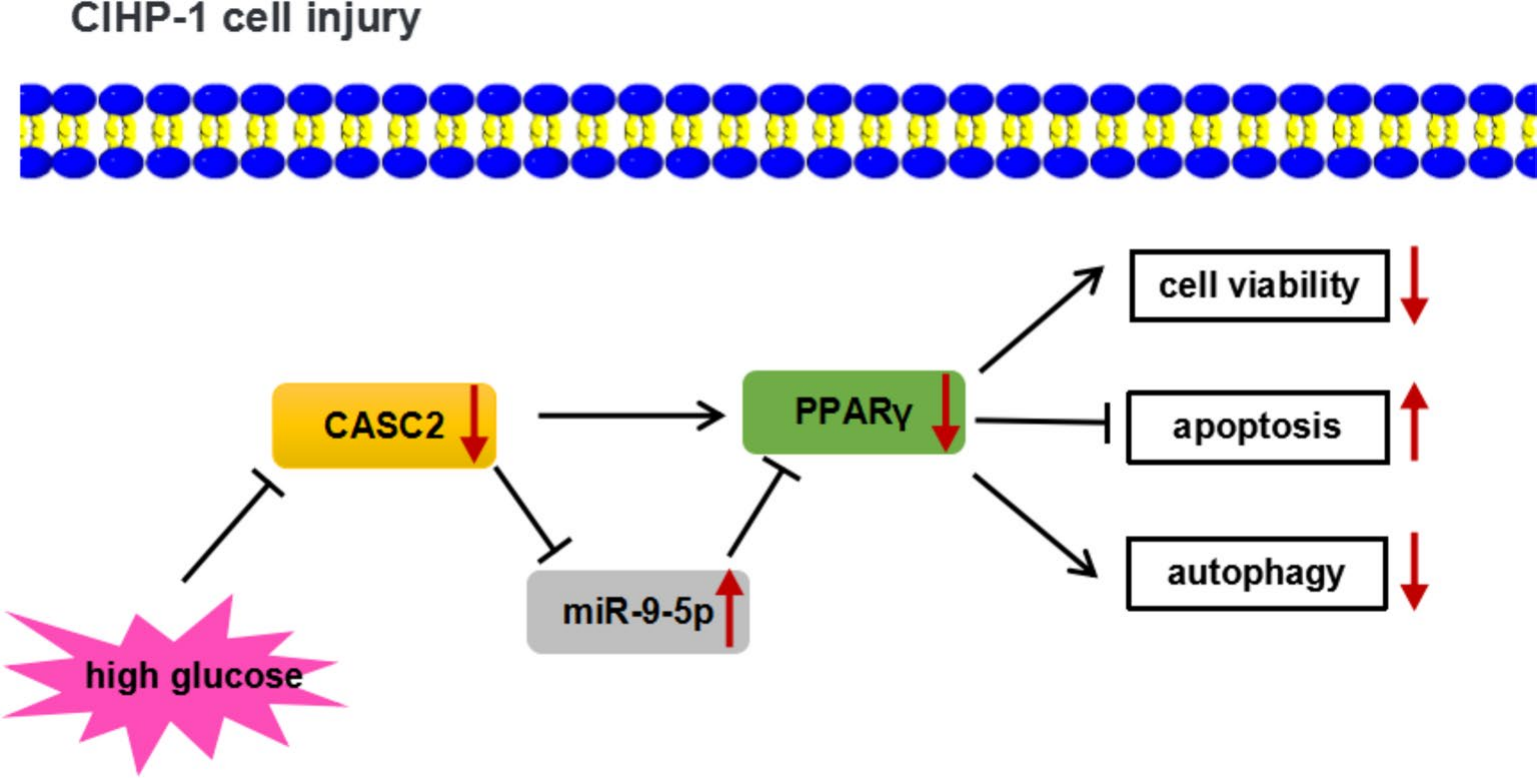
Schema presented the mechanism that HG repressed cell viability, autophagy and promoted cell apoptosis by regulating the CASC2/miR-9-5p/PPARγ axis in CIHP-1 cells

Overall, it could be concluded that HG inhibited cell viability, autophagy but promoted cell apoptosis by downregulating CASC2 and PPARγ expression as well as upregulating miR-9-5p in CIHP-1 cells (Fig. 6).

## Discussion

Podocytes are terminally differentiated visceral epithelial cells, which are important components of the glomerular filtration barrier. Podocyte viability and apoptosis as well as autophagy can affect glomerular function [21]. A large number of studies have shown that high glucose induction can cause podocytes injury [22–24].

Several lncRNAs, such as lncRNA MALAT1 [25] and lncRNA PRINS [26], have been found to be involved in the development of DN, they regulated mRNA expression at the post-transcriptional level. In this study, we found that CASC2 expression was prominently down-regulated in high glucose-stimulated podocytes in a time-dependent manner and dose–response manner. Autophagy is a double-edged sword, and its excessive activation or repression can cause podocytes injury [27]. Autophagy activity is impaired in DN patients, so promoting autophagy to some extent can reduce podocytes injury [28], and Beclin 1, LC3-I and LC3-II have been shown to be autophagy specific proteins [29]. In accordance with previous data, high glucose could inhibit cell viability and autophagy, and promote cell apoptosis, while overexpression of CASC2 could attenuate the effect of high glucose on podocytes injury, suggesting the protective effect of CASC2 on podocytes injury. Similarly, Yang et al. observed that CASC2 was enormously decreased in DN patients, while there was no significant difference in CASC2 expression in DN patients as compared to those with DM without complication (DN) [30]. Besides, Wang et al. reported that the development of type 2 diabetes might have no significant effects on CASC2 expression in renal tissue, whereas CASC2 expression in renal tissues was found to be evidently lower in patients with type 2 diabetes complicated with chronic renal failure [13]. These data suggested that CASC2 inhibition was very likely to be involved in the pathogenesis of DN. Moreover, lncRNA often functions as ceRNA, and we speculated that CASC2 might also be involved in the regulation of podocytes injury by sponging miRNA.

Compared with non-diabetic subjects, the level of miR-9-5p was higher in serum of patients with gestational diabetes mellitus [31], and serum miR-9 might be an underlying marker for poor prognosis of DN [32]. In our data, the abundance of miR-9-5p was increased in high glucose-induced podocytes, and miR-9-5p was validated to be the target miRNA for CASC2, and CASC2 could inversely modulate miR-9-5p expression in podocytes. Recovery experiments showed that CASC2 mitigated podocytes injury by decreasing miR-9-5p via acting as a miR-9-5p sponge, similar to the work of Zhang et al., who indicated that lncRNA SOX2OT could reduce the high glucose-stimulated podocytes damage by autophagy induction through binding to miR-9 [33]. Therefore, it is reasonable to infer that CASC2 regulated podocyte activity, apoptosis and autophagy through sponging miR-9-5p.

PPARγ agonists have been widely reported to improve glycemic status in diabetes patients [34], and PPARγ has favorable renal protective effects [35]. As expected, high glucose treatment obviously retarded PPARγ expression. Importantly, miR-9-5p directly targeted PPARγ 3′UTR and negatively modulated its expression. In addition, CASC2 could regulate PPARγ expression by sponging miR-9-5p, based on these results, we hypothesized whether CASC2 implicated in podocytes injury by regulating PPARγ. The results showed that PPARγ knockdown neutralized the effect of CASC2 on podocytes injury. Besides, PPARγ has been shown to restore podocyte integrity to improve proteinuria [36].

## Conclusion

In summary, we believed that CASC2 mainly up-regulated the expression of PPARγ by acting as the ceRNA of miR-9-5p, thus alleviating HG-induced podocytes injury through increasing cell viability, autophagy and reducing cell apoptosis. This study provided a new molecular regulatory mechanism for podocytes injury induced by HG in DN.

## Data Availability

The datasets used and/or analyzed during the current study are available from the corresponding author on reasonable request.

## Abbreviations

HG: High glucose
DN: Diabetes nephropathy
CASC2: Cancer susceptibility candidate 2
NG: Normal glucose
RIP: RNA immunoprecipitation
PPARγ: Peroxisome proliferator-activated receptor gamma
